# DA-BioNER: data augmentation based on few-shot learning and distant supervision for biomedical named entity recognition

**DOI:** 10.1093/bioinformatics/btag332

**Published:** 2026-05-22

**Authors:** Yesol Park, Gyujin Son, Taeuk Kim, Mina Rho

**Affiliations:** Department of Computer Science, Hanyang University, Seoul, Republic of Korea; Department of Artificial Intelligence, Hanyang University, Seoul, Republic of Korea; Department of Computer Science, Hanyang University, Seoul, Republic of Korea; Department of Artificial Intelligence, Hanyang University, Seoul, Republic of Korea; Department of Computer Science, Hanyang University, Seoul, Republic of Korea; Department of Artificial Intelligence, Hanyang University, Seoul, Republic of Korea; Department of Biomedical Informatics, Hanyang University, Seoul, Republic of Korea

## Abstract

**Motivation:**

Named entity recognition (NER) is a fundamental component of structured knowledge extraction, yet its effectiveness in emerging domains remains by the scarcity of high-quality, domain-specific annotated corpora. Although data augmentation and distant supervision have been explored to alleviate this issue, existing methods often introduce limited entity diversity, noisy labels, or disrupt contextual integrity, thereby limiting their generalization ability in low-resource settings.

**Results:**

In this study, we propose DA-BioNER, a context-preserving data expansion framework for biomedical NER. DA-BioNER combines multiple base NER models trained on few-shot data to provide coarse annotations, followed by refinement using a large language model (LLM) guided by global biomedical knowledge. Unlike generation-based augmentation methods that synthesize new sentences, DA-BioNER performs annotation refinement within existing sentences, preserving both syntactic structure and semantic context. By constraining the role of LLM to refinement rather than open-ended generation, the framework effectively reduces hallucination while improving label precision and consistency. We evaluate DA-BioNER on three benchmark datasets (NCBI-Disease, BC5CDR, and BioRED), under low-resource conditions. In 40-shot settings, DA-BioNER achieves F1-scores of 0.750, 0.795, and 0.799, respectively, outperforming state-of-the-art methods, including LSMS, DAGA, and MELM, by up to 0.32. Under more extreme few-shot settings, DA-BioNER further improves F1-scores by up to 0.08, while generating an average of 1,391 additional unique entities, substantially enriching training diversity. These results demonstrate that DA-BioNER provides a scalable and adaptable solution for robust biomedical NER, particularly in domain adaptation and low-resource scenarios.

**Availability:**

DA-BioNER is publicly available at https://github.com/DMnBI/DA-BioNER.

## 1 Introduction

Named entity recognition (NER) is a core task in natural language processing (NLP), involving the identification and classification of semantically meaningful entities—such as personal names, organizations, or domain-specific terms—within unstructured text (Catelli*et al.* 2020, Sun*et al.* 2021, Asghari*et al.* 2022). In the biomedical domain, where terminology is dense and specialized, accurate NER is indispensable for transforming free-text literature into structured knowledge. This capability supports a wide range of downstream applications, including relation extraction, semantic search, knowledge graph construction, and hypothesis generation (Luo *et al.* 2020, Perera *et al.* 2020, Rossanez *et al.* 2020, Harnoune *et al.* 2021).

Despite its central role in biomedical text mining, the development of high-performance NER models remains constrained by the scarcity of annotated corpora. Although fine-tuning pre-trained language models (PLMs) can reduce the amount of data required for domain adaptation, high-quality biomedical corpora are still limited. Constructing such datasets is both costly and time-consuming, requiring substantial domain expertise. Consequently, existing biomedical datasets are often small and narrowly focused, typically covering only a limited range of entity types, such as diseases, genes/proteins, chemicals, and species (Košprdić *et al.* 2024).

Large language models (LLMs), such as GPT (Brown *et al.* 2020), T5 (Raffel *et al.* 2020), and Llama (Touvron *et al.* 2023), have recently demonstrated remarkable generalization and reasoning capabilities across various NLP tasks. Unlike PLMs, which are fine-tuned for specific downstream tasks, LLMs can perform complex tasks through prompting alone. This flexibility has encouraged efforts to apply LLMs to biomedical NER, aiming to mitigate data scarcity and improve adaptability (Keloth *et al.* 2024, Monajatipoor *et al.* 2024, Munnangi *et al.* 2024, Lu *et al.* 2025). However, several challenges hinder their direct application. First, prompt engineering—the design of effective instructions to guide LLMs—demands significant expertise, particularly in the biomedical contexts with complex terminology and abbreviations. Second, the hallucination problem remains critical. LLMs may generate non-existent entities or assign inappropriate entity types. Finally, the high computational cost of LLM inference limits their practicality for large-scale biomedical text processing.

To address these limitations, researchers have increasingly explored data-centric approaches, particularly data augmentation, to synthetically expand training corpora while maintaining contextual and semantic integrity (Shorten *et al.* 2021, Dai *et al.* 2025). A range of augmentation techniques has been proposed, including token-level manipulation (Dai and Adel 2020), sentence-generation models like DAGA (Ding *et al.* 2020), and targeted perturbation methods such as MELM (Zhou *et al.* 2022), BioAug (Ghosh *et al.* 2023), and styleNER (Chen *et al.* 2021). More recently, LLM-driven data augmentation frameworks (e.g., LLM-DA (Ye *et al.* 2024)) have leveraged prompt-based rewriting to generate synthetic examples. However, these methods still face several challenges. Many rely heavily on limited annotated data, constraining entity diversity and novelty. Heuristic-based transformations, such as synonym substitution or mention shuffling, often introduce semantic drift and boundary inconsistencies. For example, token-level substitutions may fragment entity mentions (e.g., replacing “*muscle tear*” with “*muscle*”), degrading linguistic coherence and label accuracy. These issues are particularly problematic in biomedical contexts, where terminology precision and contextual integrity are crucial.

An alternative and complementary strategy is distant supervision, which automatically generates weakly labeled data by aligning raw text with external knowledge bases (Liang *et al.* 2020, Wang *et al.* 2020a, Wang *et al.* 2020b). Although this approach introduces label noise, it enables large-scale corpus generation without manual annotation. Moreover, recent advances in contrastive learning have demonstrated that models can still benefit from weak supervision, even in the presence of labeling errors (Li *et al.* 2021).

In this work, we present DA-BioNER, a novel framework that integrates distant supervision, few-shot learning, and LLM-guided annotation refinement to generate high-quality labeled data for biomedical NER. DA-BioNER comprises two main components: (1) multiple base NER models trained on few-shot datasets that provide coarse entity annotations; and (2) an LLM that aggregates and refines these annotations using its extensive linguistic and biomedical knowledge. By combining the scalability of NER models with the broad knowledge of LLMs, DA-BioNER generates context-preserving, diverse, and accurate training data. This framework not only alleviates data scarcity but also establishes a scalable and adaptable foundation for robust biomedical NER in low-resource domains.

## 2 Materials and methods

### 2.1 Dataset

In this work, DA-BioNER was developed and evaluated under the assumption of a data-scarce environment, which we refer to as a few-shot scenario. Specifically, we conducted experiments with 10-, 20-, 30-, and 40-shot settings, where *k*-shot denotes that *k* entities are included in the training data. The number of entities for each few-shot setting is presented in Table 1.

**Table 1.** Distribution of entities by entity type across few-shot settings.

Datasets	Entity Types	Shot			
		10-shot	20-shot	30-shot	40-shot
NCBI-Disease	Disease	10	20	31	42
BC5CDR	Disease	12	21	33	43
	Chemical	10	21	30	44
BioRED	Gene	10	20	33	46
	Disease	12	20	32	44
	Chemical	12	25	37	49
	Variant	10	25	33	41
	Species	12	22	30	46
	Cell Lines	11	23	31	40

For training and testing our framework, NCBI-Disease (Doğan *et al.* 2014), BC5CDR (Li *et al.* 2016), and BioRED (Luo *et al.* 2022) datasets were used for the biomedical domain. The NCBI-Disease includes only disease entities within biomedical literature. BC5CDR, in contrast, contains annotations for both chemical and disease entities, enabling joint entity recognition tasks. BioRED extends this further by offering a comprehensive biomedical corpus annotated with multiple entity types, including diseases, chemicals, genes, sequence variants, species, and cell lines.

### 2.2 DA-BioNER framework

DA-BioNER consists of two primary stages: (1) annotating raw text using multiple base NER models trained on a few-shot dataset, and (2) employing an LLM-based ensemble to aggregate and refine their outputs, thereby improving overall labeling quality and consistency.

#### 2.2.1  Base NER models

On the *k*-shot samples (k = 10, 20, 30, and 40), NER models were trained using transfer learning, with each model employing a distinct PLM as its backbone. These NER models utilize a sequence labeling approach. Specifically, given a sequence of n tokens $S=\{w_{1},w_{2},\ldots ,w_{n}\}$, the NER model outputs a corresponding label sequence $Y=\{y_{1},\;y_{2},\;\ldots ,\;y_{n}\}$, where each $y_{i}$ belongs to the label set L. The entity types recognized by the NER model are defined as $T=\{t_{1},t_{2},\;\ldots ,\;t_{m}\}$, and the label set L is constructed as $L=\{O\}\cup \{B_{t},\;I_{t}\;|\;t\in T\}$. Here, “O” represents a non-entity token, $“B_{t}”$ and $“I_{t}”$ indicate the first word and the other words included in each entity of type t, respectively.

#### 2.2.2  LLM-based ensemble process

**Figure 1:**
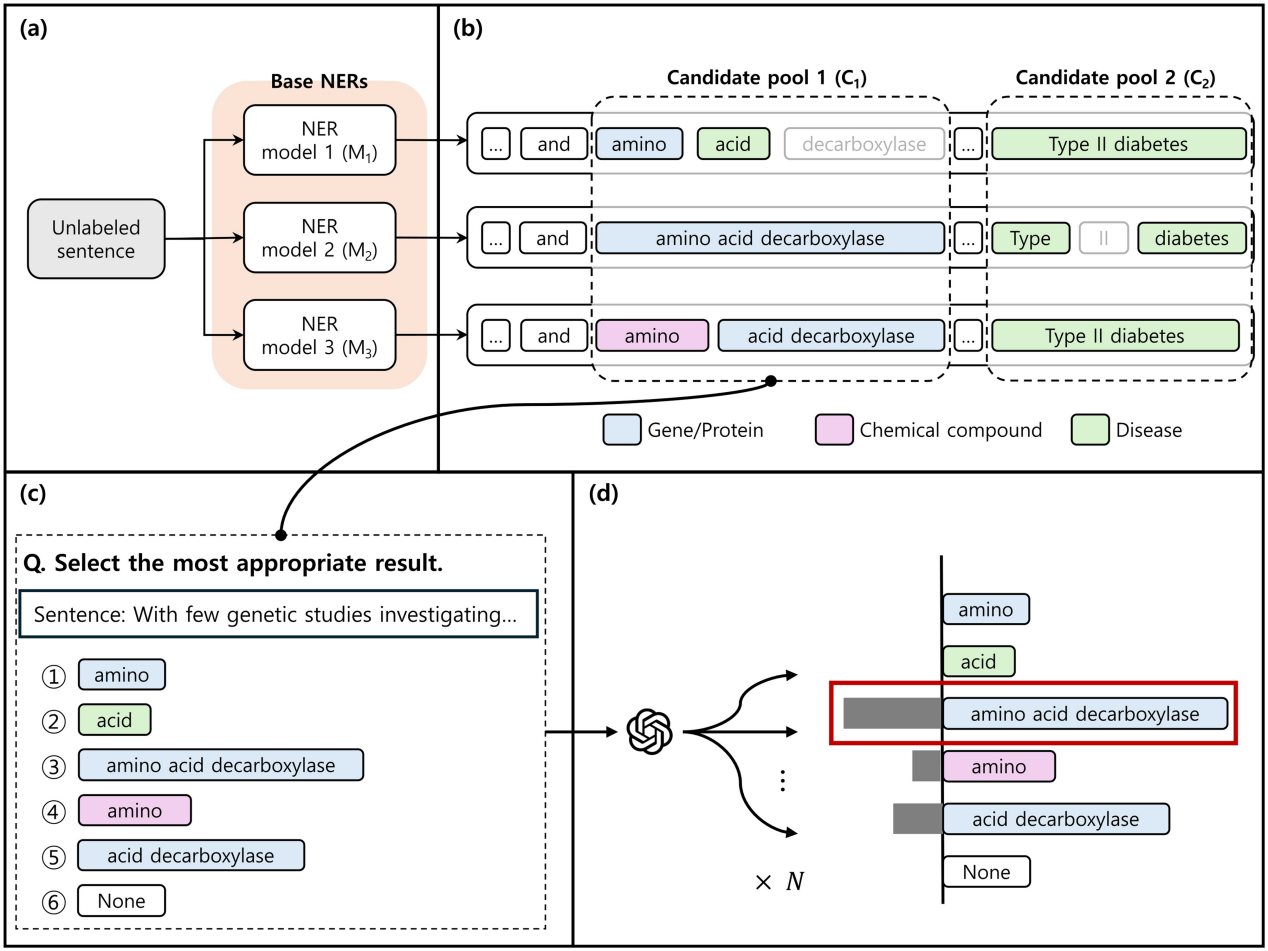
Workflow of DA-BioNER. (a) Prediction of source entities. The base models of named entity recognition (NER) independently predict the entities from unlabeled text. (b) Generation of candidate pools. The outputs from the base NER models are combined, with overlapping entities grouped together to form candidate pools. (c) Construction of candidate pools as single-select problems. The candidate pool is structured as a single-selection problem. Note that the option ‘None’ is included in the selection choices to account for instances where no suitable entity exists. This single-selection problem, along with the sentence containing the candidate pool, is then passed to the next stage. (d) Refinement of candidate pools via a large language model (LLM). The single-selection problem is input into the LLM multiple times, with the final result generated through majority voting on the outcomes.

The process of generating distant labeling is composed of four stages, as illustrated in Figure 1. Initially, entities are predicted from the unlabeled sentences using multiple base NER models (Figure 1a). The outputs from the base NER models are aggregated to construct candidate pools (Figure 1b). In this process, entities composed entirely of special characters are then filtered out to eliminate unnecessary computations. For each NER model $M_{i}$, a set of predicted entities $E_{i}\;$is generated as follows:


(1) $$E_{i}=\{\left(s_{i1},e_{i1},l_{i1}),\;(s_{i2},e_{i2},l_{i2}),\;\ldots ,\;(s_{im},e_{im},\;l_{im}\right)\},$$

where $s_{ij}$, $e_{ij}$, and $l_{ij}$ denote the start index, end index, and label (e.g., disease) of the j-th entity by the model $M_{i}$, respectively. Given multiple models $\left\{M_{1},\;M_{2},\;\ldots ,\;M_{n}\right\}$, their respective entity predictions $\left\{E_{1},\;E_{2},\;\ldots ,\;E_{n}\right\}$ are restructured to a set of candidate pools $C=\{C_{1},\;C_{2},\;\ldots ,\;C_{k}\}$. Each candidate pool $C_{i}$ is a set of entities that share overlapping spans predicted from multiple base NER models. For example, as shown in Figure 1b, the entities ‘amino’ (Gene/Protein) and ‘acid’ (Disease) from model 1, ‘amino acid decarboxylase’ (Gene/Protein) from model 2, and separate entities ‘amino’ (Chemical) and ‘acid decarboxylase’ (Gene/Protein) from model 3 are all grouped into a candidate pool because their spans overlap. A candidate pool contains only one entity when there are no overlapping entities. The overlap condition is defined as:


(2) $$\begin{array}{c} O\mathrm{verlap}(\left(s,e,l),\;(s^{\prime },e^{\prime },l^{\prime }\right))=\\ \text{True},\;\text{if}\;s\leq e^{\prime }\;\text{and}\;e\geq s^{\prime } \end{array}$$

Then, each candidate pool is formatted as a single-select problem for the verification process (Figure 1c). The selection choices include the option ‘None’ to account for instances where no suitable entity exists. In this process, entities composed of a mention and a label from the candidate pool, along with a sentence containing them, are provided to an LLM.

Utilizing this information, the LLM selects the most appropriate option (Figure 1d). This process, called the refinement process, leverages contextual understanding and an extensive knowledge base from the LLM to assess the validity and suitability of candidate entities. The detailed prompt is provided in Table 2. To ensure robust and reliable results, the refinement process is conducted multiple times for each candidate pool, with a majority voting strategy used to determine the final output. This approach helps mitigate the impact of variability in individual assessment outcomes.

**Table 2.** Example of prompt designed for ensemble and refinement.

Prompt	Given the input consisting of a sentence, biomedical entity candidates with their classes, select the most reasonable entity candidate if there is one. The selection criteria are that the candidate’s word must be valid, and the assigned class must be appropriate for the candidate. Do not modify the candidate’s word or class. If no suitable candidate is found, return no entity. Provide an explanation for the decision. Return the result in JSON format with the following fields: explanation, text, and class. The text and class fields in the output must exactly match the text and class of the selected candidate. The selected entity must be one of the provided candidates or none if no suitable candidate exists. {example}
Input	-SENTENCE: “{sentence}” -CANDIDATES: 1. “{entity mention 1}” ({entity type 1}) 2. “{entity mention 2}” ({entity type 2}) … n. “{entity mention n}” ({entity type n})

The “{example}” is substituted with examples in the same format as the input. The “{sentence}” is replaced with the original sentence to be processed. “{entity mention *i*}” and “{entity type *i*}” are replaced with the respective entity mention and type.

### 2.3 Experimental settings

**Figure 2:**
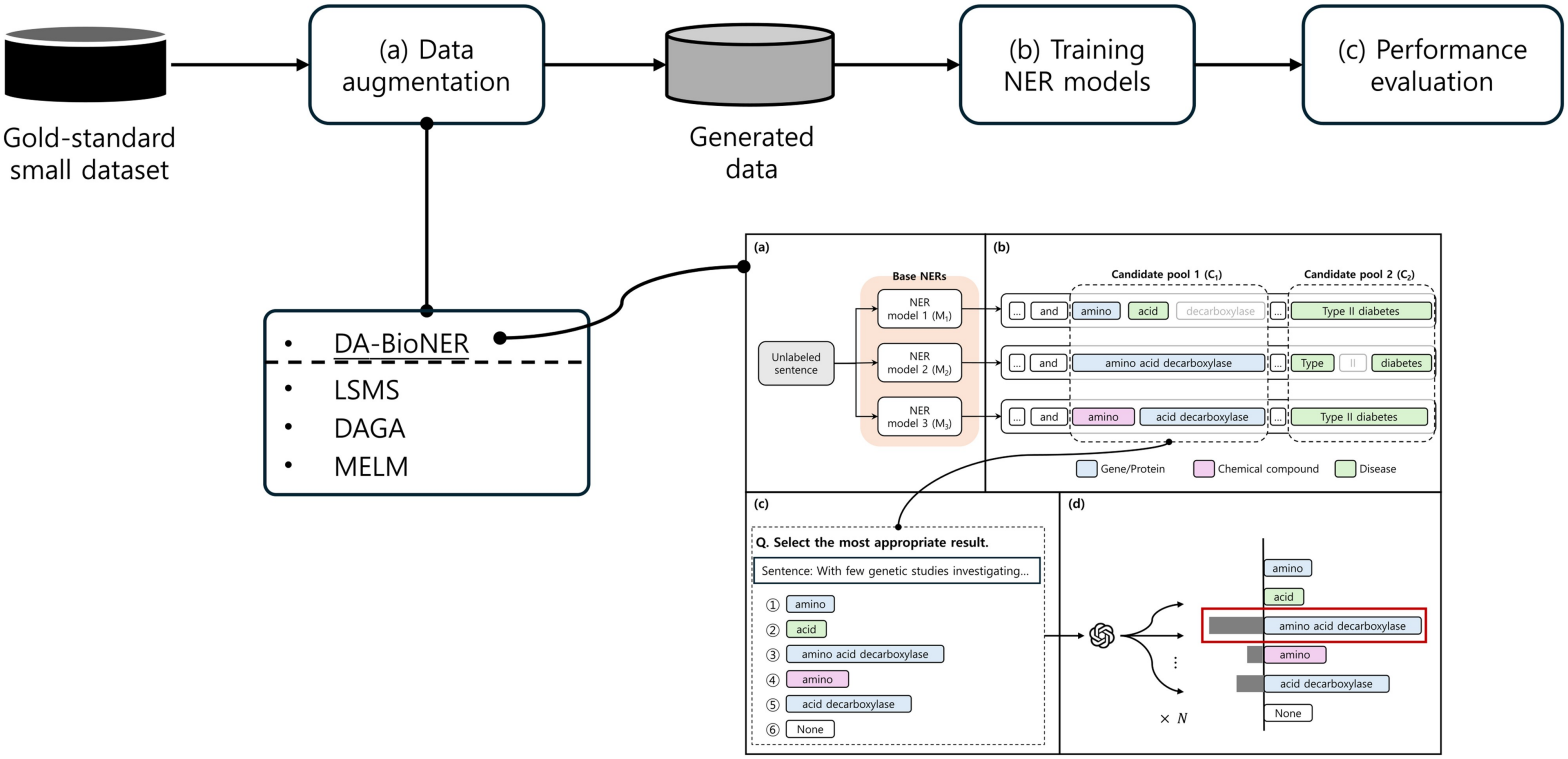
Overall process for evaluating augmented data. (a) Data augmentation. DA-BioNER or existing methods generated new data based on gold-standard data-based few-shot settings. (b) Training NER models. Independent NER models are trained on the data generated by each augmentation method. (c) Performance evaluation. Performance of the NER models are evaluated.

Figure 2 illustrates the evaluation process for the augmented data. To assess the impact of data expansion, we trained independent NER models and evaluated their performance on the corresponding test sets. The following subsections provide a detailed description of the experimental setup.

#### 2.3.1  Preparing training data and unlabeled corpora

In this study, we utilized *k*-shot samples as the training data for the data augmentation models. In the context of NER, a k-shot sample typically refers to a dataset containing exactly k entities for each class (Yang and Katiyar 2020). However, real-world data often fails to meet such strict criteria due to imbalanced class distributions. To address this issue, a flexible k-shot sampling method is applied, as proposed in (Ye *et al.* 2024), to permit each class to include between k and 1.25k entities. Building on this method, the sampling strategy includes both k to 1.25k entities per class and k negative sentences that do not contain any entities. By including negative cases in learning, the model reduces the risk of false positives (FPs), thereby preventing overfitting and improving its precision in identifying true entities.

DA-BioNER processes unlabeled text as input to generate augmented data. For this purpose, we randomly selected subsets of 500, 1,000, 2,000, 3,000, and 4,000 unlabeled sentences from each dataset. Among the three datasets, BioRED includes various entity types and exhibits significant class imbalances. Therefore, to ensure reliable experiments, each type was required to contain at least 30 entities. Note that these sentences are mutually exclusive from the few-shot samples used in the training.

#### 2.3.2  Base NER model and LLM setup

The base NER models were constructed with a PLM, a linear layer, and a SoftMax function. Each model was fine-tuned on a distinct PLM: BioBERT (Lee *et al.* 2020) (biobert-base-cased-v1.2), BiomedBERT (Gu *et al.* 2021) (BiomedBERT-base-uncased-abstract-fulltext), and Bioformer (Fang *et al.* 2023) (bioformer-16L). This approach, which uses different PLMs, allows the generation of diverse candidate outputs. The differences among these PLMs are summarized in Table 3.

**Table 3:** Comparison of biomedical PLMs.

		BioBERT_Base-v1.2_	BioMedBERT	Bioformer_16L_
Corpus	PubMed	✓	✓	✓
	PMC	✓	✓	✓
	Eng. Wiki	✓	–	–
	Books	✓	–	–
Initial weight		BERT	–	–
Vocabulary		BERT	Self-made (WordPiece)	Self-made (WordPiece)
Vocabulary size		28,996	30,522	32,768
Architecture	Heads	12	12	8
	Layers	12	12	16
	Hidden size	768	768	384
	Parameters	110M	110M	43M

During the training phase, the models were optimized using a cross-entropy loss function with a learning rate of $3\times 10^{-5}$. Batch sizes were configured as 2 for the 10-shot, 4 for the 20-shot, and 8 for both the 30-shot and 40-shot settings. The maximum sequence length for all scenarios was set to 512 tokens. An early stopping strategy was applied based on training loss, with a minimum of 30 epochs and a maximum of 50 epochs, and training was halted if the loss did not significantly reduce over 5 consecutive epochs. For the ensemble stage, the gpt-3.5 model—specifically, the gpt-3.5-turbo-0125 model developed by OpenAI—was utilized with the following settings: frequency_penalty set to 0, presence_penalty set to 0, temperature set to 0.7, and top_p set to 1.

In the experiments, we utilized two examples in the prompt, which remained consistent across all datasets, as the examples served merely as a tool to define the task (see Supplementary Table S1, available as supplementary material at *Bioinformatics* online for examples). The inference stage was conducted three times, and the final result was obtained through a majority voting strategy.

#### 2.3.3  Configuration of an NER model for the validation of augmented data

To evaluate the efficiency of our method, an NER model was independently trained on the augmented datasets generated by DA-BioNER and competing methods. The NER model mentioned in this section is completely independent of the base NER model introduced in Section 2.2 and was trained solely for evaluation purposes (Figure 2). This NER model employs a transfer learning approach, utilizing a PLM with a SoftMax classifier. Given that augmented data inherently includes a certain level of noise, a noise-tolerant approach is essential for effectively training the NER model. Therefore, we employ a symmetric cross-entropy (SCE) loss function (Wang *et al.* 2019) as optimization in the NER model. SCE integrates both standard cross-entropy (CE) and reverse CE (RCE) to enhance robustness against noisy labels. Specifically, while CE penalizes incorrect predictions, RCE mitigates the over-penalization caused by mislabeled samples by also considering the divergence from the predicted distribution to the true distribution. This formulation allows the model to learn more stably in the presence of label noise. Formally, the SCE loss is defined as follows:


(3) $$L_{CE}=\;-\sum_{i=1}^{N}y_{i}\log\left({\hat{y}}_{i}\right)$$

(4) $$L_{\mathrm{RCE}}=-\sum_{i=1}^{N}{\hat{y}}_{i}\log\left(y_{i}\right)$$

(5) $$\mathrm{SCE}=\alpha \cdot \;L_{CE}+\beta \cdot L_{\mathrm{RCE}},$$

where N is the total number of entity classes, and $y_{i}$ and ${\hat{y}}_{i}$ represent ground truth and predicted probability for class i, respectively. α and β are hyperparameters that control the contribution of the CE and RCE losses to the overall SCE loss.

The NER models were trained on the augmented datasets, with the gold standard few-shot data used for validation. For the SCE loss, the parameters were set as follows: α=0.2 and β=1.0. The learning rate was set to $5\times 10^{-5}$, with a batch size of 32 and a maximum sequence length of 512 tokens. An early stopping strategy based on validation loss was applied, with a minimum of 15 epochs and a maximum of 60 epochs, and training was terminated if the loss did not show a significant reduction over 5 consecutive epochs.

### 2.4 Comparison models

DA-BioNER was evaluated against several baselines and augmentation-based methods, including Gold-only, LSMS (Dai and Adel 2020), DAGA (Ding *et al.* 2020), MELM (Zhou *et al.* 2022), and simple voting. The Gold-only baseline model utilizes only the original dataset manually annotated without any data augmentation. LSMS generates augmented data by applying token-level transformations to entity mentions. DAGA utilizes an LSTM-based text generation model that produces synthetic sequences incorporating linearized entity labels. MELM employs a masked entity language model that selectively masks tokens within entity spans and predicts replacements to create new entity mentions. The simple voting method uses a rule-based strategy to select a representative entity label from a pool of candidates (see Supplementary Methods, available as supplementary material at *Bioinformatics* online for detailed algorithms).

The comparison models expand the data based on few-shot samples according to the following configurations. For LSMS and DAGA, approximately 4,000 augmented sentences were generated using random seeds, and subsets of 500, 1,000, 2,000, 3,000, and 4,000 sentences were randomly selected from these. For MELM, the generation process was performed only three times per input sentence, as this was identified by the authors as the optimal number of repetitions. The erroneous sentences, including blank tokens, were removed to ensure data quality. The simple voting operates in a process nearly similar to DA-BioNER, but differs in the selection strategy employed to ensemble the candidate pool.

### 2.5 Evaluation metrics

In these experiments, the models are evaluated using three metrics: micro-averaged precision, recall, and F-score. Precision and recall are defined as:


(6) $$\mathrm{precision}=\frac{\mathrm{TP}}{\mathrm{TP}+\mathrm{FP}},\;\mathrm{recall}=\frac{\mathrm{TP}}{\mathrm{TP}+\mathrm{FN}}.$$

Here, TP, or true positive, represents the number of labeled entities for which the model correctly identifies both the boundaries and the class; FP, false positive, indicates the number of incorrectly identified named entities; and FN, false negative, refers to the number of actual named entities that the model fails to identify.

The F1-score is a comprehensive metric that balances precision and recall, calculated as:


(7) $$F1=\frac{2\times \mathrm{precision}\times \mathrm{recall}}{\mathrm{precision}+\mathrm{recall}}\;.$$

In the context of NER, micro-averaging was adopted to calculate these metrics across all entities collectively, rather than averaging the metrics for each class separately.

## 3 Results and analysis

### 3.1 Performance evaluation and comparison

We evaluated the performance of DA-BioNER against state-of-the-art data augmentation methods and baseline models by training an NER model on augmented datasets and assessing its performance across a range of experimental settings (Figure 2; Table 4). DA-BioNER was implemented in two variants: DA-BioNER (SI), which performs a single round of an LLM inference, and DA-BioNER (MI), which performs three rounds of inference followed by majority voting. Comparative augmentation methods included LSMS (Dai and Adel 2020), DAGA (Ding *et al.* 2020), and MELM (Zhou *et al.* 2022), while baseline models comprised one trained solely on gold-standard annotations and a simple ensemble using majority voting.

**Table 4:** Performance comparison with existing data augmentation methods.

	NCBI-Disease (F1-score)				BC5CDR (F1-score)				BioRED (F1-score)			
	10-shot	20-shot	30-shot	40-shot	10-shot	20-shot	30-shot	40-shot	10-shot	20-shot	30-shot	40-shot
Gold-only	0.292	0.516	0.588	0.611	0.552	0.648	0.696	0.726	0.494	0.634	0.715	0.749
LSMS	**0.454**	0.626	0.657	0.687	0.658	0.699	0.756	0.779	0.655	0.708	0.737	0.764
DAGA	0.031	0.492	0.628	0.600	0.052	0.605	0.693	0.759	0.074	0.503	0.718	0.687
MELM	0.217	0.245	0.387	0.425	0.301	0.332	0.536	0.514	0.472	0.426	0.521	0.626
Simple vote	0.262	0.539	0.649	0.682	0.627	0.635	0.681	0.736	0.651	0.693	0.747	0.768
DA-BioNER (SI)	0.319	**0.650**	**0.732**	0.740	0.660	**0.727**	0.767	**0.798**	0.726	**0.761**	**0.792**	0.796
DA-BioNER (MI)	0.325	0.623	0.725	**0.750**	**0.666**	0.722	**0.773**	0.795	**0.731**	0.759	0.791	**0.799**

**Bold** indicates the best performance, and underline represents the second-best one.

Each model was evaluated across 20 distinct settings, combining five augmentation sizes (500, 1,000, 2,000, 3,000, and 4,000 sentences) with four few-shot levels (10, 20, 30, and 40 shots). All experiments were repeated three times with independently initialized random seeds, and the average F1-score was reported. The same set of random seeds was used across all models to ensure fairness. Following the assumption and approach in (Ye *et al.* 2024) that the optimal augmentation size varies with data sparsity and task complexity, we evaluated performance across five augmentation sizes and reported the highest F1-score (Table 4). For LSMS, DAGA, Simple vote, DA-BioNER (SI), and DA-BioNER (MI), we considered augmentation sizes of 500, 1,000, 2,000, 3,000, and 4,000 sentences per k-shot setting. For MELM, the augmented data was generated to be three times the size of the input sentences, following the original configuration. In addition, samples containing errors (e.g., blank tokens) are filtered out during preprocessing. Detailed performance evaluation results of DA-BioNER across all experimental settings are provided in Supplementary Table S2, available as supplementary material at *Bioinformatics* online.

As shown in Table 4, both DA-BioNER variants consistently outperformed competing methods across most settings. Under the 40-shot, DA-BioNER (MI) achieved F1-scores of 0.750, 0.795, and 0.799 on NCBI-Disease, BC5CDR, and BioRED, respectively. DA-BioNER (SI) achieved similarly competitive scores of 0.740, 0.798, and 0.796. Notably, DA-BioNER outperformed other augmentation models in most scenarios, with the exception of the 10-shot setting on NCBI-Disease. In general, DA-BioNER exceeded the best alternative by margins ranging from 0.008 to 0.076 across three datasets.

The performance gap between DA-BioNER (MI) and DA-BioNER (SI) is minimal (< 0.01 in most cases), indicating that LLM-based predictions are highly stable and robust to inference variability, even without repeated inference. Furthermore, both variants consistently outperform simple voting, suggesting that the observed performance gains are not solely due to aggregation, but are largely driven by LLM inference to select reliable entities through contextual reasoning.

### 3.2 Analysis of the quality of augmented data

**Figure 3:**
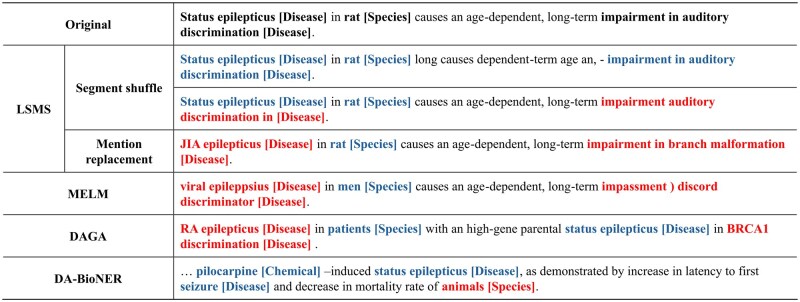
Examples of augmented sentences generated by data augmentation methods. Predicted entities are in **bold**; incorrect predictions (false positives and false negatives) are marked in red, and correct predictions (true positives) in blue.

We conducted a qualitative evaluation of augmented sentences generated by each model under the 40-shot setting on BioRED (Figure 3). For LSMS (Dai and Adel 2020) and MELM (Zhou *et al.* 2022), which apply one-to-one transformations based on input sentences, examples were selected based on the input-output sentence pairs. For DAGA (Ding *et al.* 2020) and DA-BioNER, which operate independently on input templates, examples were randomly selected from the generated corpus.

LSMS (Dai and Adel 2020) applies segment-level shuffling, which frequently results in grammatically incorrect or semantically incoherent outputs. For example, the non-entity phrase “cause an age-dependent, long-term” was distorted into “long causes dependent-term age an, -,” rendering the sentence unintelligible. Such distortions are typical of shuffling-based augmentation methods, which frequently disrupt semantic coherence and sentence fluency. Similarly, disease entities like “impairment in auditory discrimination” were shuffled into “impairment auditory discrimination in,” compromising grammatical structure.

While LSMS with mention replacement and MELM (Zhou *et al.* 2022) preserve sentence-level structure, they often introduce malformed entities by generating incorrect or artificial word combinations. MELM, for example, generated non-existent terms such as “epileppsius” and “impassment,” stemming from its token-level masking and reconstruction approach.

DAGA (Ding *et al.* 2020), combining issues from both pattern-based and generative methods, generated semantically invalid sentences with unrelated entities (e.g., “RA epilepticus [Disease]” and “BRCA1 discrimination [Disease]”). These nonsensical phrases reduce the utility of the augmented data for NER training.

In contrast, DA-BioNER generated syntactically diverse yet grammatically and semantically valid sentences. It maintains contextual integrity while generating a high proportion of correctly labeled, biologically meaningful entities—e.g., “pilocarpine [Chemical],” “status epilepticus [Disease],” and “seizure [Disease]." This balance between sentence structure and entity quality supports effective training of robust NER models.

### 3.3 Diversity of augmented entities

Entity diversity is a key factor in effective data augmentation, particularly in low-resource settings. To quantitatively assess this, we analyze generated entities by measuring both the number of *unique entities* (i.e., distinct entities after normalization) and *novel entities* (i.e., those with no lexical overlap with entities in the training data) under few-shot settings on NCBI-Disease, BC5CDR, and BioRED (Table 5). For a fair comparison, all methods generate 4,000 sentences, and the resulting entities are analyzed accordingly. MELM is excluded due to its inability to generate a sufficient number of sentences.

**Table 5:** Unique and novel entity counts from data augmentation.

		# Unique Ent. (# Novel Ent.)			
		10-shot	20-shot	30-shot	40-shot
NCBI-Disease	Gold-only	9 (-)	18 (-)	27 (-)	38 (-)
	LSMS	133 (31)	577 (31)	746 (31)	976 (31)
	DAGA	352 (298)	1,256 (298)	632 (289)	852 (289)
	DA-BioNER	539 (479)	885 (466)	1,176 (457)	1,222 (449)
BC5CDR	Gold-only	19 (-)	37 (-)	57 (-)	78 (-)
	LSMS	156 (65)	407 (65)	569 (64)	680 (63)
	DAGA	369 (286)	563 (279)	768 (279)	462 (279)
	DA-BioNER	1,349 (1,323)	1,845 (1,308)	2,186 (1,293)	2,251 (1,276)
BioRED	Gold-only	19 (-)	37 (-)	57 (-)	78 (-)
	LSMS	940 (34)	1,214 (70)	1,531 (55)	1,658 (34)
	DAGA	570 (388)	1,487 (415)	1,013 (393)	2,392 (379)
	DA-BioNER	3,455 (3,259)	3,845 (3,083)	3,987 (2,996)	4,106 (2,911)

To identify unique entities, we apply a normalization pipeline consisting of lowercasing, removal of special characters, lemmatization, and deduplication. Novel entities are then identified by applying the same preprocessing steps to the training set and selecting entities composed entirely of tokens that do not appear in the processed training set.

As shown in Table 5, DA-BioNER consistently generated the largest number of unique and novel entities, except for the NCBI-Disease 20-shot setting, where it produced fewer than DAGA. Specifically, on average, DA-BioNER generates 265, 1,411, and 2,498 more unique entities, and 301, 1,128, and 2,841 more novel entities than LSMS and DAGA across NCBI-Disease, BC5CDR, and BioRED, respectively.

To further analyze these results, we examine the augmented entities generated by each method. In the 10-shot setting of BioRED, the original set includes gene entities such as “FASN,” “Let-7b,” “TCF,” “Hsp27,” “BDNF,” “Wnt,” “MSST1,” and “KRAS.” Based on these, LSMS generated entities such as “7 b Let -” (Gene), “BDNF - -” (Gene), and “Wnt shock 7 b” (Gene), which largely reflect surface-level recombinations of observed tokens rather than semantically valid gene entities. DAGA produced entities such as “Let and” (Gene), “BDNF” (Species), “Wnt - -” (Chemical), and “BCNU rat” (Chemical), many of which are semantically invalid or assigned incorrect entity types. This suggests that increased diversity does not necessarily correspond to meaningful semantic expansion.

In contrast, DA-BioNER generates entities such as “p27” (Gene), “TCF1” (Gene), and “GSK-3beta” (Gene), which are semantically plausible and consistent with the target entity type. Moreover, it generates not only simple entities but also more complex biomedical entities, such as “Peroxisomal proliferator - activated receptor-gamma” (Gene) and “Na-K-2Cl cotransporter” (Gene). Although DA-BioNER occasionally produced malformed entities (e.g., “- 26 b” and “- alpha”), its outputs overall demonstrate more meaningful semantic expansion beyond the training distribution.

This ability of DA-BioNER to synthesize domain-appropriate and previously unseen entities is especially valuable in low-resource settings, where training data often lack sufficient coverage of the full biomedical entity space. These results demonstrated that DA-BioNER not only maintains syntactic and semantic validity but also enhances entity diversity, supporting the development of more generalizable and domain-aware NER models. Furthermore, these outcomes were achieved without relying on the inherent creativity of LLMs, as DA-BioNER deliberately constrains the role of LLM within a controlled generation framework. This design approach avoids uncontrolled hallucinations and allows the LLM to generate diverse yet domain-consistent entities.

### 3.4 Effects of LLM-based ensemble on performance

In this section, we analyze the contribution of the LLM-based ensemble approach. For the experiment, we apply multiple base NER models to a standard corpus to generate annotations, aggregate these outputs into a candidate pool, and then refine the pool using either simple voting or DA-BioNER.

When comparing the number of candidate spans, each pool contains an average of 1.06 to 1.42 candidate spans (see Supplementary Table S3, available as supplementary material at *Bioinformatics* online). Although many pools contain a single candidate, a substantial proportion includes multiple candidates—particularly in BioRED—indicating the presence of overlapping and ambiguous predictions. This highlights the importance of effective aggregation and refinement strategies.

Table 6 presents the performance evaluation results of these methods. Simple voting generally improved recall over individual base NER models, suggesting diversity among the base NER models and the benefit of aggregating their results. However, this gain in recall came at the cost of decreased precision due to noise from misaligned or spurious entity predictions.

**Table 6:** Performance evaluation of three base NER models and two ensemble results.

		10-shot			20-shot			30-shot			40-shot		
		P	R	F	P	R	F	P	R	F	P	R	F
NCBI-Disease	BioBERT	0.401	0.131	0.198	0.520	0.291	0.373	0.596	0.500	0.544	0.643	0.633	0.638
	BiomedBERT	0.462	0.120	0.191	0.632	0.509	0.564	0.645	0.649	0.647	0.633	0.640	0.637
	Bioformer	0.354	0.187	0.245	0.484	0.479	0.481	0.579	0.643	0.609	0.663	0.697	0.679
	Simple vote	0.361	0.249	0.295	0.522	0.559	0.540	0.554	**0.706**	0.621	0.602	**0.743**	0.665
	**DA-BioNER**	**0.614**	**0.250**	**0.356**	**0.722**	**0.571**	**0.637**	**0.745**	0.698	**0.721**	**0.769**	0.734	**0.751**
BC5CDR	BioBERT	0.674	0.221	0.333	0.608	0.453	0.519	0.597	0.515	0.553	0.625	0.563	0.593
	BiomedBERT	0.702	0.494	0.580	0.624	0.645	0.634	0.619	0.712	0.662	0.701	0.762	0.730
	Bioformer	0.533	0.349	0.421	0.511	0.589	0.547	0.589	0.713	0.645	0.664	0.711	0.686
	Simple vote	0.574	**0.557**	0.565	0.505	**0.685**	0.582	0.556	**0.753**	0.640	0.634	**0.789**	0.703
	**DA-BioNER**	**0.763**	0.522	**0.620**	**0.738**	0.649	**0.691**	**0.758**	0.728	**0.743**	**0.799**	0.763	**0.781**
BioRED	BioBERT	0.515	0.470	0.491	0.524	0.528	0.526	0.611	0.609	0.610	0.636	0.641	0.638
	BiomedBERT	0.589	0.561	0.574	0.653	0.689	0.670	0.689	0.730	0.709	0.722	0.767	0.744
	Bioformer	0.551	0.585	0.568	0.567	0.678	0.617	0.665	0.716	0.690	0.709	0.766	0.736
	Simple vote	0.537	**0.647**	0.587	0.580	**0.723**	0.644	0.659	**0.767**	0.709	0.690	**0.801**	0.742
	**DA-BioNER**	**0.732**	0.626	**0.675**	**0.749**	0.701	**0.724**	**0.787**	0.742	**0.764**	**0.798**	0.763	**0.780**

* **Bold** indicates the best performance, and underline represents the second-best one.

In contrast, the DA-BioNER achieved balanced improvements in both precision and recall across most tasks, with F1-score gains ranging from 0.04 to 0.11 over the best base models. Compared to simple voting, our model showed a slightly lower recall but substantially improved precision, producing consistently higher F1-scores.

**Figure 4:**
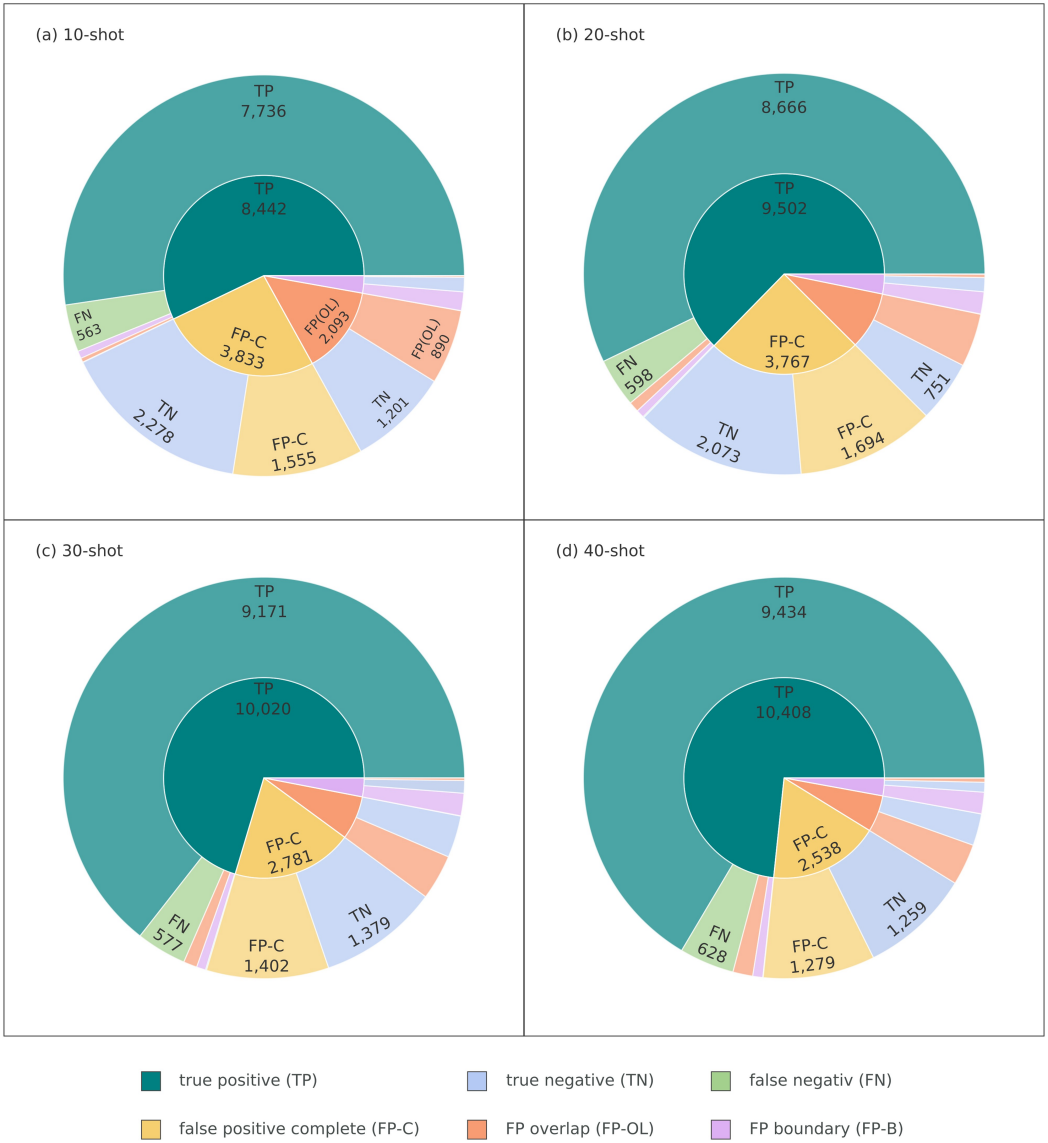
Classification metrics for the candidate pools and LLM ensemble on BioRED. The inner circle represents the classification metrics of the candidate pool aggregated from the base NER models, while the outer circle illustrates the classification metrics of the results obtained after applying the LLM ensemble. FP-C, FP-OL, and FP-B denote completely incorrect, boundary-overlapping, and boundary-matching but type-incorrect false positives (FPs), respectively. The LLM ensemble successfully removes 57.6%, 53.8%, 47.5%, and 45.9% of FPs from the candidate pool in the 10-, 20-, 30-, and 40-shot scenarios, respectively. Meanwhile, the proportion of true positives (TPs) incorrectly predicted as either false negatives (FNs) or FPs by the LLM ensemble remains low, at just 8.4%, 8.8%, 8.5%, and 9.4%, respectively.

To further assess the refinement capacity of the DA-BioNER, we compared entity prediction quality before and after ensemble refinement using BioRED (Figure 4). Metrics were computed for two stages: (1) candidate pool metrics (inner circle in Figure 4), aggregating entity predictions from all base NER models prior to ensemble, and (2) DA-BioNER metrics (outer circle), representing final entity predictions after refinement from the LLM-based ensemble model.

In the candidate pool, TP was defined as any pool containing at least one correctly predicted entity. FP included incorrect entity type with correct boundary (FP-B), overlapped boundary (FP-OL), and unrelated entities (FP-C). The DA-BioNER metrics followed standard evaluation metrics.

Across four few-shot levels, total FPs (i.e., the sum of FP-C, FP-OL, and FP-B) in the candidate pool were 6,338, 5,567, 4,216, and 3,784 out of 14,780, 15,159, 14,236, and 14,192 candidate entities, respectively. After applying the DA-BioNER, a substantial portion of these FP candidates were correctly removed. Specifically, the ensemble removed 3,651 (57.6%), 2,996 (53.8%), 2,004 (47.5%), and 1,735 (45.9%) FP entities, substantially improving final prediction quality.

For the TP entities, the base NER models correctly identified 8,442, 9,502, 10,020, and 10,408 entities out of 12,366 ground-truth entities, respectively. After DA-BioNER refinement, only 706 (8.4%), 836 (8.8%), 849 (8.5%), and 974 (9.4%) entities were incorrectly predicted as either FNs or FPs. These results confirm the strong filtering and refinement capability of the DA-BioNER model, enhancing annotation reliability and overall model performance.

The reduction in FPs can be partly attributed to the LLMs assigning a “None” label to candidate spans that do not correspond to valid entities, effectively converting them into TNs. This demonstrates that the performance gains arise not only from aggregation but also from the ability of LLMs to filter out invalid candidates through contextual reasoning.

### 3.5 Impact of LLM variants on DA-BioNER performance

We conducted additional experiments on BioRED to assess the sensitivity of the ensemble stage to decoding parameters. Temperature, which controls the stochasticity of LLM outputs, is varied to evaluate its effect. We compare temperatures of 0.7 and 1.2 across 10-, 20-, 30-, and 40-shot settings on BioRED (Table 7). The temperature of 0.7 consistently yields slightly higher F1-scores, although both settings produced comparable results, indicating that the ensemble decisions remain stable across different temperature settings.

**Table 7:** Effect of temperature on DA-BioNER performance.

Model	Temperature	BioRED (F1-score)			
		10-shot	20-shot	30-shot	40-shot
Gold-only	–	0.494	0.634	0.715	0.749
DA-BioNER	0.7	0.731	0.759	0.792	0.799
	1.2	0.728	0.760	0.785	0.796

Additionally, we compare the performance of DA-BioNER using GPT-3.5 and GPT-5.1 under the BioRED 10-shot setting to assess the impact of more advanced LLMs (Table 8). DA-BioNER with GPT-3.5 achieved a higher F1-score (0.731 ± 0.007) than GPT-5.1 (0.710 ± 0.010). Although GPT-5.1 yields higher precision, its lower recall results in an inferior F1-score, suggesting a more conservative prediction behavior. These results show that adopting a more advanced LLM does not necessarily improve performance, and GPT-3.5 remains an effective choice for the ensemble refinement stage.

**Table 8:** Effect of LLM variants on DA-BioNER performance.

	BioRED (10-shot)		
	P	R	F
DA-BioNER (GPT-3.5)	0.781 ± 0.011	**0.687 ± 0.019**	**0.731 ± 0.007**
DA-BioNER (GPT-5.1)	**0.791 ± 0.010**	0.643 ± 0.011	0.710 ± 0.010

**Bold** indicates the best performance.

### 3.6 Generalization of DA-BioNER

In this section, we investigate the generalization of DA-BioNER from three perspectives: robustness under distributional variation, applicability of general-purpose PLMs, and their effectiveness across different scientific domains.

First, we examine the robustness of DA-BioNER beyond commonly used biomedical NER benchmarks by conducting additional experiments on the NeuroTrialNER dataset (Doneva *et al.* 2024). Unlike widely used biomedical NER datasets derived from PubMed abstracts, NeuroTrialNER reflects clinical trial registry summaries, which often exhibit different patterns in terminology and structure.

To evaluate performance in low-resource settings, we construct 10-shot and 20-shot subsets. For evaluation, we consider six categories—DRUG, PHYSICAL, BEHAVIOURAL, SURGICAL, RADIOTHERAPY, and CONDITION—while excluding OTHER and CONTROL due to their limited relevance to domain-specific biomedical entities. We randomly select 15% of the corpus as the test set. DA-BioNER consistently improves performance over the gold-only baseline in both few-shot settings (Supplementary Table S4, available as supplementary material at *Bioinformatics* online). In the 10-shot setting, the F1-score increases from 0.459 to 0.558, while in the 20-shot setting, it increases from 0.604 to 0.611. Notably, the improvements are primarily driven by gains in precision. These results demonstrate that DA-BioNER remains effective on a different type of biomedical dataset with distinct characteristics, highlighting its generalizability within the biomedical domain.

Second, we evaluate the performance of DA-BioNER using general-purpose PLMs (BERT, Devlin *et al.* 2019) on the BioRED dataset. The results are provided in Supplementary Table S5, available as supplementary material at *Bioinformatics* online. Under the 10-shot and 40-shot settings, the original DA-BioNER achieves F1-scores of 0.731 and 0.799, respectively, while DA-BioNER with BERT achieves 0.671 and 0.679. These results indicate that domain-specific PLMs provide higher performance benefits; however, general-purpose PLMs still offer reasonable performance, suggesting that DA-BioNER remains applicable even in domains where domain-adapted models are not available.

Finally, we assess the cross-domain applicability of DA-BioNER by applying it to materials science NER tasks. The dataset used for this evaluation comprises seven entity types: inorganic material, symmetry/phase label, sample descriptor, material property, material application, synthesis method, and characterization method (Weston *et al.* 2019). Performance was assessed under both 10-shot and 40-shot settings using three PLMs specifically optimized for materials science tasks—MatSciBERT (Gupta *et al.* 2022), MaterialsBERT (Shetty *et al.* 2023), and MatBERT (Walker *et al.* 2021)—employed as the base NER models. Minimal adaptation was applied: the term “biomedical” in prompts was replaced with “materials science,” and domain-specific example sentences were provided to reflect materials science context and entity types. As shown in Supplementary Table S6, available as supplementary material at *Bioinformatics* online, the models trained on DA-BioNER-augmented datasets achieved improved performance compared to those trained on the original non-augmented data. The F1-score improved by 0.123 in the 10-shot setting, while it improved by 0.024 in the 40-shot setting. These performance gains were observed in both recall and precision.

These results demonstrate that DA-BioNER generalizes across datasets, model types, and domains with minimal modification, remaining effective under diverse data distributions and consistently improving performance. This indicates its robustness and applicability beyond biomedical NER.

### 3.7 Comparison with direct LLM-based NER

Previous studies have shown that directly applying LLMs to biomedical NER often leads to suboptimal performance, primarily due to a mismatch in task formulations (Wang *et al.* 2025). While NER is typically formulated as a sequence labeling task that assigns entity labels to each token, LLMs such as GPT are designed for text generation, creating a structural gap between the two paradigms. To empirically evaluate this, we compare DA-BioNER with a GPT-based NER under the 10-shot setting on BioRED. In this setup, the LLM is provided with a small number of example sentences with annotated entities as few-shot demonstrations.

As shown in Table 9, DA-BioNER achieves a precision of 0.781, recall of 0.687, and F1-score 0.731, whereas GPT-based NER achieves a precision of 0.459, recall of 0.654, and F1-score 0.540. The direct LLM-based approach shows consistently lower performance, with a particularly large drop in precision, indicating a substantial number of false positive predictions. This finding suggests that, although LLMs provide strong contextual understanding, their direct application to NER is less suited for precise and structured prediction tasks. In contrast, DA-BioNER integrates LLMs with supervised NER models within a structured framework, enabling more reliable filtering and refinement of candidate entities.

**Table 9:** Comparison with direct LLM-based NER on BioRED.

	BioRED (10-shot)		
	P	R	F
DA-BioNER	**0.781**	**0.687**	**0.731**
GPT-NER	0.459	0.654	0.540

**Bold** indicates the best performance.

## 4 Discussion and conclusion

DA-BioNER addresses several limitations of traditional data augmentation methods for biomedical NER. By incorporating distant supervision, the framework mitigates issues commonly associated with rule- or template-based augmentation approaches, such as grammatical errors, entity corruption, and contextual inconsistency. DA-BioNER employs a two-step augmentation strategy to enhance label reliability and diversity. First, multiple base NER models—each fine-tuned on distinct PLMs—are used to generate a wide range of candidate entity predictions from diverse semantic perspectives. Second, an LLM-based ensemble is applied to refine these predictions, improving both the annotation precision and consistency.

Experimental evaluations demonstrate that DA-BioNER consistently outperforms existing data augmentation methods across several benchmark datasets, especially under low-resource settings where labeled data are scarce. The method not only produces syntactically and semantically coherent augmented sentences but also introduces a broader range of biologically meaningful entities. These findings affirm the value of integrating few-shot learning with distant supervision and LLM-based refinement for robust data augmentation.

Despite its strong performance, DA-BioNER presents opportunities for further improvement. A primary challenge is the error propagation from the base NER models. When entities are misclassified or missed entirely, these errors are passed into the LLM refinement stage, which currently lacks mechanisms for correction or backtracking. Additionally, entities with partial boundary overlap or incorrect entity type assignments remain unresolved. Future iterations could incorporate boundary-aware learning or type-sensitive prompting strategies to more effectively handle such ambiguities, as suggested in recent literature (Kim *et al.* 2024).

Furthermore, while DA-BioNER consistently outperforms other augmentation methods, it still lags behind models trained on large-scale golden-standard datasets. This performance gap underscores the potential of hybrid approaches that combine DA-BioNER with a limited amount of high-quality human-annotated data, enabling more accurate supervision without incurring the full cost of manual labeling.

Overall, DA-BioNER demonstrates strong utility in generating high-quality and diverse training data in low-resource biomedical NER tasks. Beyond model training, the generated datasets can serve as effective pre-annotations to accelerate manual labeling workflows. This makes DA-BioNER particularly well-suited for under-resourced or emerging scientific fields where large-scale labeled corpora are limited or unavailable. By reducing reliance on extensive manual labeling and enabling scalable domain adaptation, DA-BioNER represents a promising step toward more accessible and generalizable NLP applications across a wider range of scientific fields.

Key points

- DA-BioNER is a novel distant supervision and data augmentation framework based on few-shot learning and large language model (LLM) reasoning for biomedical NER. It generates distantly supervised annotations on raw text using fine-tuned NER models, and then aggregates and refines these outputs with LLMs to produce high-quality annotated data with improved consistency and reliability.
- Since DA-BioNER operates on existing sentences, it mitigates the grammatical errors and contextual inaccuracies often observed in conventional data augmentation methods. Moreover, by combining few-shot learning with LLM-based reasoning, it generates diverse and domain-appropriate entities and achieves superior performance compared to existing approaches.
- Although DA-BioNER leverages LLMs, their roles are deliberately constrained to objective reasoning tasks. This design allows the framework to exploit the broad knowledge of LLMs while mitigating hallucination.
- DA-BioNER reduces reliance on costly manual labeling and enables scalable and domain-adaptable natural language processing (NLP) applications across a wide range of scientific fields.

## Supplementary Material

btag332_Supplementary_Data

## Data Availability

The data underlying this article were derived from publicly available datasets, including the NCBI-Disease (https://doi.org/10.1016/j.jbi.2013.12.006), BC5CDR (https://doi.org/10.1093/database/baw068), and BioRED (https://doi.org/10.1093/bib/bbac282) datasets. Details of the data processing procedures are described in the Methods section.

## References

[btag332-B1] Asghari M , Sierra-Sosa D, Elmaghraby AS. Biner: a low-cost biomedical named entity recognition. Inform Sci 2022;602:184–200.

[btag332-B2] Brown TB , Mann B, Ryder N et al Language models are few-shot learners. In: Proceedings of the 34th International Conference on Neural Information Processing Systems. Vancouver, Canada: Curran Associates Inc.; 2020. Article 159.

[btag332-B3] Catelli R , Gargiulo F, Casola V et al Crosslingual named entity recognition for clinical de-identification applied to a covid-19 italian data set. Appl Soft Comput 2020;97:106779. 33052197 10.1016/j.asoc.2020.106779 PMC7544600

[btag332-B4] Chen S , Aguilar G, Neves L et al Data augmentation for cross-domain named entity recognition. Online and Punta Cana, Dominican Republic. Association for Computational Linguistics, 2021, 5346–56.

[btag332-B5] Dai H , Liu Z, Liao W et al Auggpt: leveraging chatgpt for text data augmentation. IEEE Trans Big Data 2025;11:907–18. 10.1109/tbdata.2025.3536934

[btag332-B6] Dai X , Adel H. An analysis of simple data augmentation for named entity recognition. Barcelona, Spain (Online): International Committee on Computational Linguistics, 2020, 3861–3867.

[btag332-B7] Devlin J , Chang M-W, Lee K et al Bert: Pre-training of deep bidirectional transformers for language understanding. In: *Proceedings of the 2019 conference of the North American chapter of the association for computational linguistics: human language technologies, volume 1 (long and short papers).* 2019, 4171-86.

[btag332-B8] Ding B , Liu L, Bing L et al Daga: Data augmentation with a generation approach for low-resource tagging tasks, *Online* 2020. p. 6045-57. Association for Computational Linguistics.

[btag332-B9] Doğan RI , Leaman R, Lu Z. Ncbi disease corpus: a resource for disease name recognition and concept normalization. J Biomed Inform 2014;47:1–10. 24393765 10.1016/j.jbi.2013.12.006 PMC3951655

[btag332-B10] Doneva SE , Ellendorff T, Sick B et al Neurotrialner: An annotated corpus for neurological diseases and therapies in clinical trial registries, p.18868-90. Miami, FL: Association for Computational Linguistics, 2024.

[btag332-B11] Fang L , Chen Q, Wei C-H et al Bioformer: An efficient transformer language model for biomedical text mining. arXiv, 10.48550/arXiv.2302.01588, 2023, preprint: not peer reviewed.

[btag332-B12] Ghosh S , Tyagi U, Kumar S et al Bioaug: Conditional generation based data augmentation for low-resource biomedical ner. In: *Proceedings of the 46th International ACM SIGIR Conference on Research and Development in Information Retrieval*, 2023, 1853-8.

[btag332-B13] Gu Y , Tinn R, Cheng H et al Domain-specific language model pretraining for biomedical natural language processing. ACM Trans Comput Healthcare (HEALTH) 2021;3:1–23.

[btag332-B14] Gupta T , Zaki M, Krishnan NA et al Matscibert: a materials domain language model for text mining and information extraction. NPJ Comput Mater 2022;8:102.

[btag332-B15] Harnoune A , Rhanoui M, Mikram M et al Bert based clinical knowledge extraction for biomedical knowledge graph construction and analysis. Comput Methods Programs Biomed Update 2021;1:100042.

[btag332-B16] Keloth VK , Hu Y, Xie Q et al Advancing entity recognition in biomedicine via instruction tuning of large language models. Bioinformatics 2024;40. 10.1093/bioinformatics/btae163 PMC11001490 38514400

[btag332-B17] Kim S , Seo K, Chae H et al Verifiner: Verification-augmented ner via knowledge-grounded reasoning with large language models, p.2441-61. Bangkok, Thailand: Association for Computational Linguistics, 2024.

[btag332-B18] Košprdić M , Prodanović N, Ljajić A et al From zero to hero: harnessing transformers for biomedical named entity recognition in zero-and few-shot contexts. Artif Intell Med 2024;156:102970. 39197375 10.1016/j.artmed.2024.102970

[btag332-B19] Lee J , Yoon W, Kim S et al Biobert: a pre-trained biomedical language representation model for biomedical text mining. Bioinformatics 2020;36:1234–40. 31501885 10.1093/bioinformatics/btz682 PMC7703786

[btag332-B20] Li J , Sun Y, Johnson RJ et al Biocreative v cdr task corpus: a resource for chemical disease relation extraction. Database 2016;2016:baw068. 27161011 10.1093/database/baw068 PMC4860626

[btag332-B21] Li P , Wang J, Qiao Y et al An effective self-supervised framework for learning expressive molecular global representations to drug discovery. *Brief Bioinform* 2021;22:bbab109. 10.1093/bib/bbab109 33940598

[btag332-B22] Liang C , Yu Y, Jiang H et al Bond: Bert-assisted open-domain named entity recognition with distant supervision. In: *Proceedings of the 26th ACM SIGKDD international conference on knowledge discovery & data mining.* Virtual Event, CA, USA: ACM, 2020, 1054–64.

[btag332-B23] Lu Q , Li R, Wen A et al Large language models struggle in token-level clinical named entity recognition. In: *AMIA Annual Symposium Proceedings*. San Francisco, CA, USA: American Medical Informatics Association, 2025. Abstract 2024. p.748. PMC12099373 40417588

[btag332-B24] Luo L , Lai P-T, Wei C-H et al Biored: a rich biomedical relation extraction dataset. Briefings in Bioinformatics 2022;23:bbac282. 35849818 10.1093/bib/bbac282 PMC9487702

[btag332-B25] Luo L , Yang Z, Cao M et al A neural network-based joint learning approach for biomedical entity and relation extraction from biomedical literature. J Biomed Inform 2020;103:103384. 32032717 10.1016/j.jbi.2020.103384

[btag332-B26] Monajatipoor M , Yang J, Stremmel J et al Llms in biomedicine: A study on clinical named entity recognition. *arXiv preprint arXiv : 240407376,*https://doi.org/10.48550/arXiv.2404.07376, 2024, preprint: not peer reviewed.

[btag332-B27] Munnangi M , Feldman S, Wallace B et al On-the-fly definition augmentation of llms for biomedical ner, p.3833-54. Mexico City, Mexico: Association for Computational Linguistics, 2024.

[btag332-B28] Perera N , Dehmer M, Emmert-Streib F. Named entity recognition and relation detection for biomedical information extraction. Front Cell Dev Biol 2020;8:673. 32984300 10.3389/fcell.2020.00673 PMC7485218

[btag332-B29] Raffel C , Shazeer N, Roberts A et al Exploring the limits of transfer learning with a unified text-to-text transformer. J Mach Learn Res 2020;21:1–67.34305477

[btag332-B30] Rossanez A , Dos Reis JC, Torres R et al Kgen: a knowledge graph generator from biomedical scientific literature. BMC Med Inform Decis Mak 2020;20:314–24. 33317512 10.1186/s12911-020-01341-5 PMC7734730

[btag332-B31] Shetty P , Rajan AC, Kuenneth C et al A general-purpose material property data extraction pipeline from large polymer corpora using natural language processing. NPJ Comput Mater 2023;9:52. 37033291 10.1038/s41524-023-01003-w PMC10073792

[btag332-B32] Shorten C , Khoshgoftaar TM, Furht B. Text data augmentation for deep learning. J Big Data 2021;8:101. 34306963 10.1186/s40537-021-00492-0 PMC8287113

[btag332-B33] Sun C , Yang Z, Wang L et al Biomedical named entity recognition using bert in the machine reading comprehension framework. J Biomed Inform 2021;118:103799. 33965638 10.1016/j.jbi.2021.103799

[btag332-B34] Touvron H , Lavril T, Izacard G et al Llama: Open and efficient foundation language models*arXiv preprint arXiv : 230213971,* https://doi.org/10.48550/arXiv.2302.13971, 2023 preprint: not peer reviewed.

[btag332-B35] Walker N , Trewartha A, Huo H et al The impact of domain-specific pre-training on named entity recognition tasks in materials science. *Available at SSRN 3950755* 2021. 10.1016/j.patter.2022.100488 PMC9024010 35465225

[btag332-B36] Wang S , Sun X, Li X et al Gpt-ner: Named entity recognition via large language models. In: *Findings of the association for computational linguistics: NAACL 2025*. Albuquerque, New Mexico: Association for Computational Linguistics, 2025, p.4257–75.

[btag332-B37] Wang X , Guan Y, Zhang Y et al Pattern-enhanced named entity recognition with distant supervision. In: *2020 IEEE International Conference on Big Data (Big Data)*. Atlanta, GA, USA (Virtual Event): IEEE, 2020a, 818–27.

[btag332-B38] Wang X , Song X, Li B et al Fine-grained named entity recognition with distant supervision in covid-19 literature. In: *2020 IEEE International Conference on Bioinformatics and Biomedicine (BIBM)*. Seoul, South Korea (Virtual Conference): IEEE, 2020b, 491–4.

[btag332-B39] Wang Y , Ma X, Chen Z et al Symmetric cross entropy for robust learning with noisy labels. In: *Proceedings of the IEEE/CVF international conference on computer vision*. Seoul, South Korea: IEEE, 2019, 322–30.

[btag332-B40] Weston L , Tshitoyan V, Dagdelen J et al Named entity recognition and normalization applied to large-scale information extraction from the materials science literature. J Chem Inf Model 2019;59:3692–702. 31361962 10.1021/acs.jcim.9b00470

[btag332-B41] Yang Y , Katiyar A. Simple and effective few-shot named entity recognition with structured nearest neighbor learning. *Online*. p.6365-75. Association for Computational Linguistics, 2020.

[btag332-B42] Ye J , Xu N, Wang Y et al Llm-da: Data augmentation via large language models for few-shot named entity recognition*arXiv preprint arXiv : 240214568,* 10.48550/arXiv.2402.14568, 2024, preprint: not peer reviewed.

[btag332-B43] Zhou R , Li X, He R et al Melm: Data augmentation with masked entity language modeling for low-resource ner. p.2251-62. Dublin, Ireland: Association for Computational Linguistics, 2022.

