# Supplementary material for: DA-BioNER: data augmentation based on few-shot learning and distant supervision for biomedical named entity recognition
Source: Bioinformatics. 2026 May 22;42(6):btag332. doi: 10.1093/bioinformatics/btag332 (PMC13275125; doi:10.1093/bioinformatics/btag332)
Supplement: btag332_Supplementary_Data [file btag332_supplementary_data.docx]

**Supplementary Materials and Methods**

**DA-BioNER: data augmentation based on few-shot learning and distant supervision for biomedical named entity recognition**

Yesol Park^1^, Gyujin Son^2^, Taeuk Kim^1,2^, Mina Rho^1,2,3,†^

^1^Department of Computer Science, Hanyang University, Seoul, Republic of Korea; ^2^Department of Artificial Intelligence, Hanyang University, Seoul, Republic of Korea; ^3^Department of Biomedical Informatics, Hanyang University, Seoul, Republic of Korea

† To whom correspondence should be addressed.

**Supplementary Section S1. Experimental setting for MELM**

Based on their methodology, we used XLM-RoBERTa-base as the language model. For training MELM, the batch sizes were set to 8 for the 10-shot, 16 for the 20-shot and 30shot, and 32 for 40-shot scenario, in accordance with the number of sentences in each few-shot dataset. Given the limited sample of the training data, the number of epochs was set to 60. The learning rate was set to $1\times{10}^{-5}$, as recommended by the authors. Following their approach, we similarly initialized the embeddings of label tokens using token embeddings that are semantically related the label names. Specifically, <B-disease> and <I-disease> were initialized with the embedding of the token “disease,” <B-gene> and <I-gene> with “gene,” <B-chemical> and <I-chemical> with “chemical,” <B-species> and <I-species> with “species,” and <B-variant> and <I-variant> with “variant.” In contrast, for <B-cellline> and <I-cellline>, we used the embedding of the token “cell,” as the token “cellline” was not present in the tokenizer’s vocabulary. In addition, sentences with tokens incorrectly predicted as null (“”) were removed for error handling.

**Pseudocode for simple voting ensemble**

**Supplementary Table S1. Examples Used in Prompts for Experiments**

| [Example 1] INPUT: -SENTENCE: "Knockdown and overexpression of Psoriasin in pancreatic cancer cells was performed using specifically constructed plasmids , which either had anti - Psoriasin ribozyme transgene or the full length human Psoriasin coding sequence ." -CANDIDATES:  1. "full length human Psoriasin coding sequence" (Gene or gene product)  2. "human" (Organism taxon)  OUTPUT: {  "explanation": "The most reasonable candidate is 'human'. In the sentence, 'human' specifies the source organism for the Psoriasin coding sequence. Although 'full length human Psoriasin coding sequence' is a valid candidate, it is a more complex entity. Therefore, 'human' is the most appropriate choice based on the given the context of the sentence.",  "text": "human",  "class": "Organism taxon" }  [Example 2] INPUT: -SENTENCE: "The results showed that OB counteracts most of the neurotransmitters changes caused by WRS ." -CANDIDATES:  1. "WRS" (Chemical entity)  2. "WRS" (Disease or phenotypic feature)  OUTPUT: {  "explanation": "Neither of the candidates is suitable in this context. 'WRS' is not correctly classified as either a 'Chemical entity' or a 'Disease or phenotypic feature.' The sentence does not provide enough context to confirm whether 'WRS' represents a specific disease or a chemical entity. Therefore, no entity is selected.",  "text": None,  "class": None } |
| --- |

This can be applied to the prompts presented in Table 2 of the main manuscript.

**Supplementary Table S2. Detailed F1-Scores of DA-BioNER (MI) Across Experimental Settings**

| Datasets | Sentences | Shot | | | |
| --- | --- | --- | --- | --- | --- |
|  |  | 10-shot | 20-shot | 30-shot | 40-shot |
| NCBI-Disease | 500 | 29.87±2.55 | 61.25±1.55 | 71.04±1.24 | 68.95±3.4 |
|  | 1,000 | **32.46±2.3** | **62.34±2.15** | 68.27±0.83 | **74.96±0.43** |
|  | 2,000 | 28.84±1.3 | 60.27±0.5 | 72.2±0.78 | 74.18±1.32 |
|  | 3,000 | 28.62±2.3 | 60.63±1.46 | **72.47±0.45** | 74.13±0.51 |
|  | 4,000 | 27.31±2.19 | 62.29±0.65 | 71.59±0.96 | 74.39±0.67 |
|  | Max F1- score | **32.46±2.3** | **62.34±2.15** | **72.47±0.45** | **74.96±0.43** |
| BC5CDR | 500 | 66.09±0.38 | 71.03±2.24 | 75.47±0.48 | 76.91±1.42 |
|  | 1,000 | **66.56±0.48** | 70.03±1.46 | 75.71±1.2 | 79.23±0.17 |
|  | 2,000 | 65.69±0.35 | 71.61±0.69 | 76.91±0.4 | 79.54±0.27 |
|  | 3,000 | 66.36±0.58 | 71.8±0.81 | 76.25±0.34 | **79.54±0.22** |
|  | 4,000 | 65.8±0.82 | **72.19±0.62** | **77.27±0.11** | 78.64±0.21 |
|  | Max F1-score | **66.56±0.48** | **72.19±0.62** | **77.27±0.11** | **79.54±0.22** |
| BioRED | 500 | 71.16±0.74 | 74.06±0.44 | 76.8±0.77 | 73.36±4.13 |
|  | 1,000 | 71.78±0.88 | 74.59±0.87 | 78.06±0.3 | 79.37±0.24 |
|  | 2,000 | 72.33±0.42 | 75.01±0.44 | 78.17±0.55 | 79.68±1.37 |
|  | 3,000 | **73.08±0.65** | 75.58±0.04 | 78.96±1.29 | **79.85±0.48** |
|  | 4,000 | 72.59±0.32 | **75.9±0.13** | **79.15±0.74** | 79.42±0.84 |
|  | Max F1-score | **73.08±0.65** | **75.9±0.13** | **79.15±0.74** | **79.85±0.48** |

* **Bold** indicates the best performance.

**Supplementary Table S3. Number of Candidates per Candidate Pools**

| Dataset | Shot | Number of Candidates per pools | | | | |
| --- | --- | --- | --- | --- | --- | --- |
|  |  | 1 | 2 | 3 | 4+ | Avg. Candidates |
| NCBI-Disease | 10-shot | 2,374 | 150 | 60 | 2 | 1.11 |
|  | 20-shot | 3,346 | 363 | 225 | 85 | 1.27 |
|  | 30-shot | 3,886 | 545 | 262 | 60 | 1.27 |
|  | 40-shot | 3,708 | 460 | 286 | 100 | 1.30 |
| BC5CDR | 10-shot | 7,641 | 392 | 39 | 4 | 1.06 |
|  | 20-shot | 10,563 | 325 | 261 | 43 | 1.09 |
|  | 30-shot | 9,749 | 656 | 621 | 75 | 1.19 |
|  | 40-shot | 8,837 | 657 | 662 | 73 | 1.22 |
| BioRED | 10-shot | 11,531 | 2,169 | 727 | 353 | 1.33 |
|  | 20-shot | 11,312 | 2,191 | 1,121 | 535 | 1.42 |
|  | 30-shot | 10,929 | 1,953 | 932 | 422 | 1.37 |
|  | 40-shot | 11,027 | 1,911 | 870 | 384 | 1.35 |

**Supplementary Table S4. Performance of DA-BioNER on NeuroTiralNER**

|  | 10-shot | | | 20-shot | | |
| --- | --- | --- | --- | --- | --- | --- |
|  | P | R | F | P | R | F |
| Gold-only | 0.418 | 0.509 | 0.459 | 0.559 | **0.658** | 0.604 |
| DA-BioNER | **0.567** | **0.550** | **0.558** | **0.612** | 0.610 | **0.611** |

* **Bold** indicates the best performance.

**Supplementary Table S5. Performance of DA-BioNER with General-Purpose PLMs**

|  | 10-shot | | | 40-shot | | |
| --- | --- | --- | --- | --- | --- | --- |
|  | P | R | F | P | R | F |
| DA-BioNER  using BERT | 0.738 | 0.616 | 0.671 | 0.663 | 0.696 | 0.679 |
| DA-BioNER  using BioMedBERT | 0.781 | 0.687 | 0.731 | 0.792 | 0.806 | 0.799 |

Supplementary Table S6. Performance of DA-BioNER in a domain of materials sciences

|  | 10-shot | | | 40-shot | | |
| --- | --- | --- | --- | --- | --- | --- |
|  | P | R | F | P | R | F |
| Gold-only | 0.480 | 0.380 | 0.424 | 0.654 | 0.681 | 0.667 |
| DA-BioNER | **0.576** | **0.522** | **0.548** | **0.679** | **0.704** | **0.691** |

* **Bold** indicates the best performance.
